# Magnetic Resonance Imaging of Multiple Cerebral and Spinal Cavernous Malformations of a Patient with Dementia and Tetraparesis

**DOI:** 10.3390/diagnostics12030677

**Published:** 2022-03-10

**Authors:** Florian Antonescu, Ioana Butnariu, Florentina Melania Cojocaru, Daniela Nicoleta Anghel, Dana Antonescu-Ghelmez, Sorin Tuță

**Affiliations:** 1 Department of Neurology, “Carol Davila” University of Medicine and Pharmacy, 050471 Bucharest, Romania; florian_antonescu@yahoo.com (F.A.); danaghelmez@gmail.com (D.A.-G.); sorin_tt@yahoo.com (S.T.); 2 Department of Neurology, National Institute of Neurology and Neurovascular Diseases, “Carol Davila” University of Medicine and Pharmacy, 041902 Bucharest, Romania; ivanciu.florentina@yahoo.com (F.M.C.); daniela_anghel1@yahoo.com (D.N.A.)

**Keywords:** cavernoma, cerebral cavernous malformation, familial cavernomatosis, spinal cavernous malformation, dementia, tetraparesis, magnetic resonance imaging

## Abstract

Cavernomas are rare cerebrovascular malformations that usually occur in sporadic forms with solitary lesions located most often in the hemispheric white matter, but also in the infratentorial or spinal region. Multiple lesions at different CNS levels are considered a hallmark for the familial form of the disease. The diagnostic modality of choice for cerebral cavernous malformations (CCMs) is magnetic resonance imaging (MRI). We present an intriguing case of a 65-year-old male admitted to our hospital with tetraparesis and cognitive impairment where highly sensitive MRI sequences identified many cerebral cavernous lesions at the supra-, infratentorial and cervical–thoracic spine levels, some of them with recent signs of bleeding in a patient with oral anticoagulant therapy due to atrial fibrillation. The mechanism of cognitive impairment in this patient is most probably the interruption of strategic white matter tracts, as it is known to happen in other subcortical vascular pathologies. MRI can be helpful not only in mapping the anatomical distribution of lesions, but also in weighing the risks and making decisions regarding whether or not to continue oral anticoagulant therapy.

**Figure 1:**
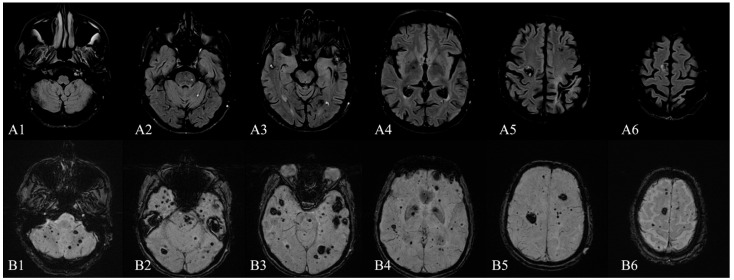
Brain MRI (1.5 T)—December 2015: (**A1**–**A6**) axial FLAIR sequence and (**B1**–**B6**) axial SWI sequence (TE 40.00 ms) showing multiple infra- and supratentorial, bilateral, cavernous malformations (lesions in the order of tens), without recent signs of bleeding. The characteristic MRI appearance of CCMs consists of well-circumscribed “popcorn-like” lesions of different sizes with a mixed-signal intensity core and a hypointense peripheral rim of hemosiderin conferring a “blooming” effect [1,2,3]. This heterogeneous appearance is due to the presence of blood products in various stages of degradation [4]. Cavernous malformations are present in 0.3–0.5% of the general population, although some postmortem studies suggest that this number could be higher, up to 4% [1,5]. In most cases, they occur in sporadic forms (80%), usually with solitary lesions, but familial cases have also been reported. Familial cases usually present with multiple, congenital, and acquired lesions, in various locations: cerebral, spinal, retinal, and cutaneous [6,7,8]. Topographically, the majority of CNS cavernous malformations have a supratentorial localization (76%), followed by infra-tentorial regions (19%), the spinal involvement being seldomly encountered (5%) [9]. Furthermore, concomitant lesions at all of the three levels are exceptionally rare and they are a particularity of the familial forms of cavernous malformations [10,11]. Clinically, the majority of cerebral cavernous malformations (CCMs) become symptomatic between the second to fifth decades with findings such as seizures (23–50% of cases), headaches (6–52%), focal neurological deficits (20–45%) or hemorrhages (9–56%) [2,9,12]. Up to 40% of patients with such malformations are asymptomatic [12]. Imaging-wise, T2-weighted, gradient-echo MR (T2-GRE), high-field MR and especially susceptibility-weighted (SWI) MR sequences are the most sensitive techniques for detecting CCMs [1,13]. Here, we present the case of a 65-year-old man diagnosed in 2015 in our clinic with multiple supra- and infratentorial CCMs (Figure 1), whom we were following for secondary epilepsy with rare seizures with focal onset, impaired awareness, and secondary generalization. He used to take carbamazepine, but he had discontinued the treatment for the last three years despite our recommendations. More recently, he had been receiving a direct-acting oral anticoagulant indicated by the cardiologist as primary prophylaxis of cardioembolic stroke from atrial fibrillation with a CHA_2_DS_2_-VASc score of 2. Recently, in 2021, the patient was re-admitted into our clinic, this time for postural instability and motor deficit with gait impairment, with the onset three days prior. The initial neurologic examination showed temporo-spatial disorientation, hypophonia, right-side central facial paresis, asymmetric tetraparesis (force grading on the MRC scale of 4/5 in the upper limbs and 3/5 in the lower limbs), with the right side slightly more affected, upper limbs dysmetria, brisk deep tendon reflexes with Babinski sign, abnormal ataxic gait, which was possible for only a few steps with bilateral support. We also noted a significant cognitive decline from MMSE of 23 points in 2015 (mainly due to attention, orientation, and executive functions deficits) to severe dementia with a MMSE of 8 points in 2021, when the patient presented significantly slowed behavioral reaction time with failure to initiate Trail Making Test part B, reasoning difficulties and forgetfulness.

**Figure 2:**
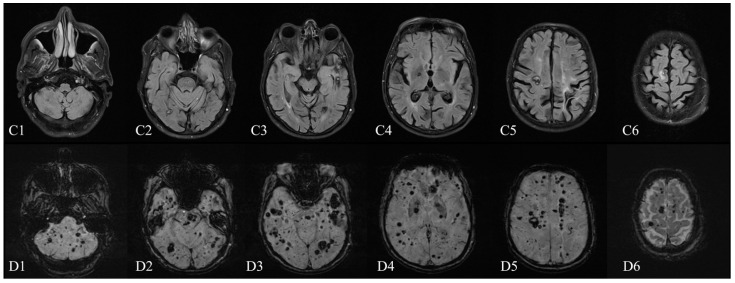
Brain MRI (1.5 T)—January 2021: (**C1**–**C6**) axial FLAIR sequence and (**D1**–**D6**) axial SWI sequence (TE 40.00 ms). Compared with the 2015 MRI (1.5 T), the number and the dimensions of cavernomas substantially increased in almost every cerebral location (comparing the B1–B6 to the D1–D6 SWI series of imaging). The 2021 cerebral MRI (Figure 2) showed a significant increase in dimensions of many CCMs as well as the addition of novel lesions compared with the previous examination, without signs of acute bleeding. Some of the large CCMs (over 10 mm) identified on the 2015 MRI examination, of the Zabramski I and II subtype, had evolved to the Zabramski III subtype (Figure 3); moreover, countless new Zabramski type IV small lesions were observed [14]. The spinal examination revealed multiple focal lesions at cervical and thoracic levels, also highly suggestive of cavernous malformations (Figure 4C). One of the lesions, at the C3–C4 level, showed signs of recent hemorrhage (Figure 4A,B).

**Figure 3:**
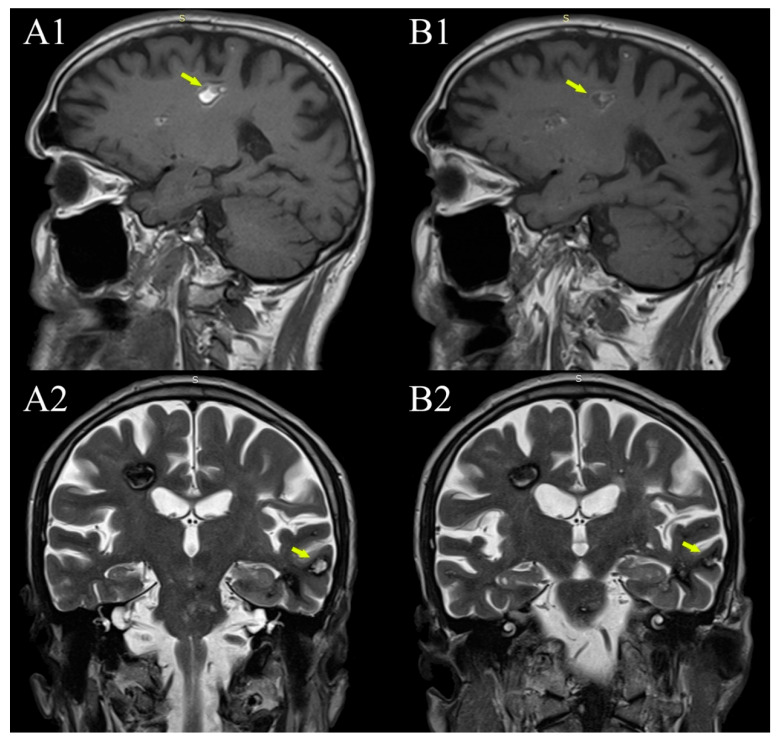
Evolution of in-cavernomas bleeding over time. (**A1**,**A2**) are from the 2015 MRI examination (1.5 T), (**B1**,**B2**) are from the 2021 examination (1.5 T). Coexistence of subacute bleeding in the right parietal lobe white matter cavernoma with T1 hyperintense signal (**A1**—arrow) and T2 inhomogeneous hyper- and hypointense signal and acute bleeding in a smaller left temporal lobe cavernoma with hyperintense T2 signal (**A2**—arrow) (but also hyperintense T1, not shown here). Both lesions became more hypointense in both T1 and T2 on the 2021 MRI examination (**B1**,**B2** —arrows).

**Figure 4:**
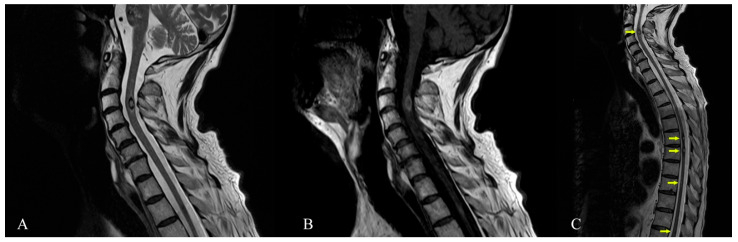
Cervical spine MRI (1.5 T)—January 2021. (**A**) Sagittal T2-TSE sequence showing intramedullary cavernoma at C3–C4 level with thin peripheral T2 hypointense ring—suggestive for gliotic sequelae changes secondary to a small bleeding cavernoma. (**B**) Sagittal T1-TSE intramedullary cavernoma at C3–C4 level (**C**) Cervical thoracic spine MRI, from January 2021, sagittal T2-TSE sequence showing focal lesions (arrows) with mixed-signal, located intramedullary at C3 level (dimensions of 8/5/7 mm), T6 level (dimensions about 5/4.5/6 mm), T7 level (dimensions of approximately 4.5/4.5/6 mm), T9 level (maximum diameter of approximately 3 mm), T11 and T12 level (two millimetric lesions)—suggestive MRI appearance for spinal cavernomas. Familial cerebral cavernous malformation syndrome (FCCM) is an autosomal dominant disease caused by the presence of a mutation in one of three known genes: KRIT-1 (CCM-1), CCM-2, and PDCD-10 (CCM-3) [15]. To diagnose FCCM one of the following three criteria has to be met: the presence of multiple CCM (typically 5 or more) or the occurrence of CCM in at least two members of a family or the presence of a mutation in one of the three genes causing FCCM [6]. For our patient, the diagnosis can be established by the very high number of cavernous malformations and the numerical progression over time. Unfortunately, despite our best effort, the patient refused genetic testing and none of the family members accepted to undergo an MRI examination. The patient was finally transferred to a rehabilitation clinic with improved tetraparesis and autonomous walking. The severe cognitive impairment persisted unabated. The risk of cavernomas bleeding under oral anticoagulants is not fully evaluated and the current scarce evidence allows for contradictory opinions [16,17] . We took into consideration the patient’s recent spinal cavernous malformation bleeding, the large brainstem lesions and the score of 3 on the cavernoma grading system, with a 55% chance of unfavorable outcome (especially for patients with a history of Zabramski I and II lesions). Additionally, bearing in mind the CHA_2_DS_2−_VASc score of 2, we were led to the conclusion that the haemorragic risk of the numerous cavernous malformations (especially brainstem or spinal lesions) outweighed the potential benefits of the oral anticoagulant therapy and the treatment was stopped [9,18]. Generally, asymptomatic cavernous malformations are only observed with serial MRI, with surgical resection reserved for some specific instances if they become symptomatic (accessible brain lesions can be surgically resected especially if they cause progressive deficits or intractable epilepsy) [19]. Spinal lesions are more difficult to treat in this way, but positive results have been reported especially with microsurgery and neurophysiological intraoperative monitoring [20,21]. In our patient, the number of brain lesions or the location of some of them (cervical spinal cord) made resection attempts impractical or with a very high risk. The case was debated with our neurosurgery team and the consensus was against an intervention for the spinal lesions. Stereotactic radiosurgery is an alternative in patients with inaccessible lesions, but with high complication rates; thus, the decision must be individualised [22,23]. We refrained from stereotactic radiosurgery as the concern exists that radiation therapy may promote the development of new lesions in familial cases [19]. The patient was convinced to resume the antiepileptic treatment with Levetiracetam. The cognitive impairment associated with multiple cavernous malformations is not a commonly recognized cause of progressive dementia. There are not many publications about multiple cavernous malformations, and among them those related to associated dementia are even rarer. In our review of the literature, we found only three published cases of cavernous malformations and cognitive deterioration [24,25,26]. Two patients of 72 and 73 years had extrapyramidal signs: one had prominent cerebellar signs and the other one some degree of hydrocephalus. None had seizures. The third reported case was of a young man of 34 years with seizures and cognitive decline, but who also had superficial siderosis on cerebral MRI. Only one of the cases was followed longitudinally with MRI and, similarly to our own, the number and size of lesions significantly increased over time. One of the limitations of this clinical case presentation is the lack of the beta-amyloid cerebral imaging, which although helpful was not available. The possibility that the patient had an overlapping of amyloid pathology and subcortical vascular lesions (cavernous malformations), both contributing to cognitive decline, cannot be ruled out. We want to make an argument in favor of subcortical vascular mechanisms of dementia in this case by referring to another, much similar, subcortical vascular pathology, much more studied than cavernomas. In this respect, small vessel disease (including lacunes, white matter lesions, cerebral microbleeds) is a better known and more frequent form of vascular disease often progressing to dementia. The onset is often with executive dysfunction and reduced processing speed, like in our patient, which is not typical for Alzheimer’s disease [27]. While the etiology of epilepsy has a significant influence on cognition, there is increasing evidence that prolonged or recurrent seizures can cause or exacerbate cognitive impairment [28]. However, our patient had no seizures while under treatment and a low frequency (one seizure every five to six months) since he interrupted antiepileptic therapy. He had no history of status epilepticus. Therefore, we think epilepsy could have had only a minor contribution to cognitive decline. The cognitive impairment associated with small vessel disease has a strong correlation with the interruption of strategic white matter tracts in frontal-subcortical neuronal circuits, girus cinguli, anterior thalamic radiation, forceps minor, medial-basal temporal and mid-occipital lobes, basal ganglia and internal capsule [29,30,31,32]. Our patient, through the sheer number of cavernomas, had diffuse involvement in almost all these regions and therefore his cognitive decline may be explained through disruption of these complex subcortical–cortical brain networks. Additionally, we think there was a possibility of damage to the medial and lateral cholinergic pathways that run forward to the orbitofrontal white matter, where he also had cavernomas [33]. As the presented images show, the degree of parenchymal atrophy and the gliotic changes remain quite similar between the two subsequent MRI studies. Additionally, there is no significant hippocampal area atrophy. Given the severe cognitive decline of the patient, we think these can be arguments in favor of a subcortical vascular mechanism, rather than Alzheimer’s disease, as the cause for dementia. If we compare the lesions induced by cavernomas to brain subcortical networks to those produced by lacunes, our patient would have a lower risk of Alzheimer’s disease pathology as a cause of dementia based on the Seoul criteria for differentiating Pittsburgh compound B (PiB)-negative from PiB-positive subcortical vascular dementia (having much more than five significant subcortical lesions, age of only 65 years and lack of significant hippocampal atrophy) [27,34]. The widespread small type IV Zabramski CCMs of this patient do not have the lobar (near cortical-subcortical junction) pattern of distribution of cerebral microbleeds associated with amyloid angiopathy. Another recently described possibility for the association of familial multiple cavernoma and dementia is the combination of the CCM2 gene variant c.236_237delAC and the APOE-e4 homozygous status with early Alzheimer-like onset of cognitive decline [35]. In conclusion, we presented the imaging of a rare clinical case of multiple cerebral and spinal cord cavernous malformations, intriguing through the sheer number of lesions, the late onset of symptoms (the patient first became symptomatic around the age of 60) and also through the possible implication in mechanisms of dementia. MRI can provide answers not only regarding the mechanisms of dementia, but it is also useful in evaluating the risk and benefit ratio when oral anticoagulant therapy is needed. In the absence of advanced beta-amyloid cerebral imaging to prove the contrary, an overlapping with Alzheimer’s disease as a cause of dementia remains possible, however, there are significant arguments for a predominant contribution of the subcortical cavernous lesions in this case. Together with the other few similar cases published, we consider that our case could be hypothesis-generating about the possible role of multiple cavernous malformations as a cause of dementia.
